# Mechanisms of Impaired Swallowing on Thin Liquids Following Radiation Treatment for Oropharyngeal Cancer

**DOI:** 10.1044/2020_JSLHR-19-00220

**Published:** 2020-08-05

**Authors:** Carly E. A. Barbon, Douglas B. Chepeha, Andrew J. Hope, Melanie Peladeau-Pigeon, Ashley A. Waito, Catriona M. Steele

**Affiliations:** a Swallowing Rehabilitation Research Laboratory, KITE, Toronto Rehabilitation Institute, University Health Network, Ontario, Canada; b Department of Speech-Language Pathology, Rehabilitation Sciences Institute, University of Toronto, Ontario, Canada; c Princess Margaret Cancer Centre, University Health Network, Toronto, Ontario, Canada

## Abstract

**Purpose:**

Dysphagia is one of the most debilitating chronic symptoms experienced by patients who undergo radiation treatment for head and neck cancer. Despite the high prevalence of dysphagia in patients with head and neck cancer, we lack understanding of the specific changes in swallowing physiology that arise in the short-term following radiation therapy and how these changes impact the key functions of swallowing safety and efficiency. This study sought to identify pathophysiological mechanisms underlying impairments in swallowing safety and efficiency on thin liquids following (chemo)radiation for oropharyngeal cancer.

**Method:**

Videofluoroscopic swallowing studies were completed in 12 male patients within 6 months following completion of (chemo)radiation and in 12 healthy male controls. To compare swallowing function and physiology between groups, we analyzed three discrete sips of 20% w/v thin liquid barium per participant. The videofluoroscopic swallowing study recordings were rated for swallowing safety, efficiency, timing parameters, and pixel-based measures of structural area or movement.

**Results:**

The oropharyngeal cancer cohort displayed significantly higher frequencies of penetration–aspiration, incomplete laryngeal vestibule closure, prolonged time-to-laryngeal vestibule closure, and poor pharyngeal constriction. Incomplete or delayed laryngeal vestibule closure was associated with airway invasion, while poor pharyngeal constriction was associated with pharyngeal residue.

**Conclusions:**

This study highlights the primary mechanisms behind impaired safety and efficiency of the swallow in patients following (chemo)radiation for oropharyngeal cancer.

Definitive radiation therapy, with or without chemotherapy, has become a first line of treatment for individuals diagnosed with oropharyngeal carcinomas (OPCs). Rather than have patients undergo surgery, radiation therapy allows for structural preservation of the head and neck. Despite advances in radiation approaches, preservation of structure does not equate to preservation of function. Radiation dose to several key anatomic structures, including the pharyngeal constrictors and larynx, has been associated with dysphagia characterized by aspiration (impaired swallowing safety) and pharyngeal residue (impaired swallowing efficiency; Eisbruch et al., 2004). Despite the high prevalence of dysphagia in head and neck cancer (HNC) patients, we lack understanding of the specific changes in swallowing physiology that arise in the short term following radiation therapy and how these changes impact the key functions of swallowing safety and efficiency.

## Factors Related to Swallowing Safety

Airway protection during the swallow involves closure of the laryngeal vestibule (LVC; Logemann et al., 1992; Vose & Humbert, 2018). Complete LVC requires elevation of the hyolaryngeal complex, arytenoid-to-epiglottic contact, and adduction of the vocal folds (Ardran & Kemp, 1952, 1967; Inamoto et al., 2011). The integrity and timing of LVC are critical for preventing penetration–aspiration (Rofes et al., 2010; Steele et al., 2015), while short duration of LVC (LVCDur) may be related to aspiration risk. The data regarding LVC after Radiation Therapy (RT) are unclear regarding whether deficits exist in the integrity, timing, or duration of closure (Kotz et al., 2004; Nativ-Zeltzer et al., 2014).

## Factors Related to Swallowing Efficiency

Pharyngeal residue is a common finding in individuals with dysphagia, and previous studies have implicated reduced pharyngeal constriction as a primary mechanism associated with pharyngeal residue (Dejaeger et al., 1997; Kahrilas et al., 1992; Langmore & Krisciunas, 2010; Stokely et al., 2015; Ursino et al., 2016; Waito, Tabor-Gray et al., 2018). Data demonstrate worse pharyngeal constriction and motility for oropharynx cancer patients treated with RT compared to patients with alternate tumor locations (Kendall et al., 1998; Kotz et al., 1999, 2004). Additional pharyngeal parameters thought to have implications for bolus transit and clearance include the area of the pharyngeal lumen at rest and the diameter and duration of upper esophageal sphincter (UES) opening. While HNC patients may display atrophic pharyngeal musculature as a late radiation effect, they are also more likely to display narrowing of the pharynx in earlier stages due to pharyngeal edema (Isitt, 2006; Murphy & Gilbert, 2009; Turcotte et al., 2018). Radiation-induced stenosis of the UES has also been reported as a negative sequela of (chemo)radiation and may be associated with pharyngeal residue (Eisbruch et al., 2002; Farwell et al., 2010; Kotz et al., 2004; Pauloski et al., 1998). The impact of these pharyngeal and UES-based changes on pharyngeal constriction and bolus clearance remains unclear.

## Research Questions

Further investigations are required to elucidate the mechanisms behind impairments of swallowing safety and efficiency in the post-(chemo)radiation HNC population. Given this gap, we sought to conduct a detailed analysis of swallowing following the completion of (chemo)radiotherapy for oropharyngeal cancer and aimed to answer the following questions regarding the swallowing of thin liquids in a prospective patient sample:

1a. What is the prevalence of impaired swallowing safety compared to that seen in healthy controls?

1b. What is the prevalence of incomplete LVC compared to that seen in healthy controls, and is it associated with penetration–aspiration?

1c. Does the timing or duration of LVC differ compared to values seen in healthy controls, and are these parameters associated with penetration–aspiration?

2a. What is the prevalence of impaired swallowing efficiency compared to that seen in healthy controls?

2b. Do measures of pharyngeal area at rest (PhAR), pharyngeal area at maximum constriction (PhAMPC), or the duration and diameter of UES opening differ from values found in healthy controls?

2c. Are measures of pharyngeal area or UES opening related to the presence of pharyngeal residue?

We hypothesized that penetration–aspiration following (chemo)radiation treatment for oropharyngeal cancer would be associated with incomplete LVC and prolonged time-to-LVC (or maximum approximation of the arytenoids to the laryngeal surface of the epiglottis) but not with abnormalities in LVC duration. We expected that oropharyngeal cancer patients would display smaller pharyngeal areas at rest (secondary to edema), larger pharyngeal areas at maximum constriction, smaller diameters of UES opening, and shorter durations of UES opening compared to healthy controls. We expected that these differences would be associated with increased pharyngeal residue.

## Method

The study was approved by the local institutional research ethics board. Participants provided written consent prior to their participation.

### Oropharyngeal Cancer Cohort

A prospective sample of patients with a primary diagnosis of OPC treated with primary RT was recruited from a clinic at a large cancer institution. Radiation oncology patient lists were reviewed weekly to identify all new and established OPC patients who met study criteria. Patients were approached to consider study participation either before, during, or after completion of their treatment, unless otherwise specified by their radiation oncologist. In total, 12 male patients with OPC consented to participate.

### Healthy Control Group

A cohort of 12 healthy control participants was selected from the data set of a larger, ongoing study exploring the physiology of swallowing in healthy adult volunteers. Control participants were identified in the available data set based on sex (males only) and the closest possible match in age.

### Data Collection

Each participant underwent a videofluoroscopic swallowing study (VFSS) performed in lateral projection at 30 pulses per second and recorded on a KayPENTAX Digital Swallow Workstation at 30 frames per second. The protocol began with a series of three discrete sips of thin liquid barium. Comfortable sips were taken from cups containing 40 ml of fluid (one sip per cup), and sip volume was derived from pre- and postsip measurements of cup weight. Participants self-administered the boluses and swallowed when ready without waiting for a cue from the clinician. Prespecified stopping rules were followed, such that further boluses of thin liquid were discontinued after the first occurrence of penetration or aspiration without ejection was noted by the clinicians present at the VFSS. This resulted in fewer than three thin liquid boluses being available for three of the OPC participants in whom Penetration–Aspiration Scale (PAS) scores of 3, 5, and 7 were immediately obvious during data collection (Rosenbek et al., 1996).

### Videofluoroscopy Rating

The VFSS recordings were rated in duplicate by trained raters using ImageJ software (National Institutes of Health) according to a standard operating procedure known as the ASPEKT Method (Analysis of Swallowing Physiology: Events, Kinematics, and Timing), which has been described elsewhere (Steele et al., 2019). Due to delays between the completion of data collection for the two study cohorts, raters were not blinded to participant group at the time of VFSS analysis. The following parameters of interest were recorded for each bolus:

1. PAS: The worst PAS score across all subswallows for each bolus was recorded.
2. LVC integrity: This was measured on the initial swallow of each bolus and classified as complete or incomplete. Cases of partial closure were classified as incomplete.
3. Timing measures for the initial swallow of each bolus are as follows:
  - Time-to-LVC: the interval between the frame of hyoid burst onset and the frame of LVC or maximum approximation of the arytenoids to the epiglottis;
  - LVCDur: the interval between the frame of LVC onset and the frame of LVC offset;
  - UES opening duration (UESDur): the interval between UES opening and UES closure.
4. Pixel-based measures are as follows:
  - Total residue: Residue was traced on the frame of swallow rest for each swallow and referenced against the squared C2–C4 spine length reference scalar (C2–4)^2^ to derive a residue percentage. In the event that multiple swallows were observed for a single bolus, the swallow rest frame exhibiting the greatest residue was selected.
  - Pharyngeal area: Pixel-based tracings were made of PhAR and on the frame of PhAMPC using the terminal swallow rest frame and initial swallow Maximum Pharyngeal Constriction frame for each bolus, respectively, and expressed as a percentage of the (C2–4)^2^ reference scalar.
  - UES diameter: The anterior–posterior width of the UES as viewed on a lateral X-ray image was measured on the frame of maximum UES distension for the initial swallow of each bolus by tracing a line perpendicular to the spine at the narrowest point between C4 and C6 (Leonard et al., 2004a) and expressed as a percentage of the C2–C4 scalar.

#### Data Reduction

Prior to statistical analysis, continuous parameter data were inspected for extreme outliers (i.e., 3 times the interquartile range below the first or above the third quartile (Rosner, 2016, p. 31), normality (assessed by Shapiro–Wilk tests), and homogeneity of variance (assessed using Levene's tests). UES diameter data were unavailable for one OPC and one healthy participant.

In order to support the subsequent statistical analysis, the following parameters were transformed into binary categorical variables:

1. Safety binary: PAS scores were dichotomized into “safe” scores of no concern (i.e., scores of 1 and 2) versus “impaired” scores (i.e., scores ≥ 3).
2. Residue binary: Total residue was dichotomized below versus ≥ 3% of the (C2–4)^2^ reference area, based on the 95th %ile for data previously reported for healthy young adults (Steele et al., 2019).
3. PhAR was dichotomized as “unusually small” versus “within normal range” using a threshold of < 36% (C2–4)^2^, based on the 5th %ile for data previously reported for healthy young adults (Steele et al., 2019). A secondary threshold of < 47% (C2–4)^2^ based on the first quartile boundary for young adults was also explored, with values between 36% and 47% (C2–4)^2^ considered “small.”
4. PhAMPC was dichotomized based on the 95th %ile from reference data for healthy young adults (Steele et al., 2019), that is, below versus ≥ 4% (C2–4)^2^.

Intraclass correlation coefficients (ICC; two-way mixed, absolute agreement) were calculated to measure interrater reliability for continuous measures; kappa scores were calculated for binary categorical measures. As shown in Table 1, interrater agreement was moderate to excellent (Koo & Li, 2016; Viera & Garrett, 2005) for all parameters. Any disagreements in rating that fell outside prespecified targets for interrater agreement were resolved by consensus.

**Table 1.** Intrarater agreement statistics for Penetration–Aspiration Scale (PAS) scores, frame selection, and pixel-based measurements.

Parameter	Level of data	Agreement statistic	Agreement value (% agreement or 95% CI)	Interpretation^a^	% Requiring consensus resolution
PAS	Nominal	Kappa	.483 (71%)	Moderate	29
LVC (complete/incomplete)	Binary	Kappa	.539 (88%)	Moderate	12
Hyoid burst frame	Continuous	ICC^b^	.996 [.995, .997]	Excellent	2
LVC frame	Continuous	ICC^b^	.996 [.994, .997]	Excellent	6
LVC offset frame	Continuous	ICC^b^	.997 [.996, .998]	Excellent	2
Maximum pharyngeal constriction frame	Continuous	ICC^b^	.998 [.997, .998]	Excellent	10
UES opening frame	Continuous	ICC^b^	.998 [.997, .998]	Excellent	2
Max UES distension frame	Continuous	ICC^b^	.998 [.997, .998]	Excellent	< 1
UES closure frame	Continuous	ICC^b^	.957 [.889, .936]	Excellent	14
Swallow rest frame	Continuous	ICC^b^	.925 [.898, .945]	Excellent	21
UES diameter	Continuous	ICC^b^	.541 [.244, .711]	Moderate	4
PhAR	Continuous	ICC^b^	.808 [.708, .869]	Good	3
PhAMPC	Continuous	ICC^b^	.900 [.863, .926]	Excellent	5
Total residue %(C2–4)^2^	Continuous	ICC^b^	.890 [.837, .924]	Good	2

*Note.* LVC = laryngeal vestibule closure; ICC = interclass correlation coefficient; UES = upper esophageal sphincter; PhAR = pharyngeal area at rest; PhAMPC = pharyngeal area at maximum constriction.

a Qualitative interpretation of agreement statistics taken from Viera and Garrett (2005) and Koo and Li (2016).

b ICC model = two-way mixed, absolute agreement.

In order to summarize performance per participant, the following rules were applied:

1. For measures of penetration–aspiration, the worst score seen across the available thin liquid boluses for each participant was used.
2. For measures capturing LVC integrity or timing, the values associated with the initial subswallow for the bolus with the bolus with the worst PAS score were used. In the event that all boluses for a participant displayed safe PAS scores of 1 or 2, LVC data for the first bolus were used.
3. For measures of PhAR, the average was calculated across all available thin liquid boluses for each participant.
4. For measures of residue, PhAMPC and UES diameter/duration, the bolus displaying the worst residue was used. In the event that all boluses for a participant displayed residue < 3% of the (C2–4)^2^ area, data for the first bolus were used.

## Analysis

Binary logistic regression and odds ratios (*OR*s) were used to investigate group differences in the frequencies of at least one bolus per participant showing impaired swallowing safety (PAS scores ≥ 3), incomplete LVC, and/or impaired swallowing efficiency (total pharyngeal residue ≥ 3% (C2–4)^2^). One-way analyses of variance were conducted to determine whether measures of timing (time-to-LVC, LVCDur, UESDur) and pixel-based measures of UES diameter differed between groups. Due to nonnormal distribution of residuals, Kruskal–Wallis *H* tests were performed to determine if there were group differences in PhAR and pharyngeal residue. Fisher's exact tests and *OR*s were used to determine mechanistic relationships between

1. binary classifications of LVC integrity (complete vs. incomplete) and swallowing safety (≤ 2 safe vs. ≥ 3 impaired);
2. binary classifications of unusually small and small PhAR (< 36% (C2–4)^2^, < 47% (C2–4)^2^) and pharyngeal residue of concern (> 3% (C2–4)^2^); and
3. binary classifications of poor pharyngeal constriction (> 4% (C2–4)^2^) and pharyngeal residue of concern (> 3% (C2–4)^2^).

Finally, in order to evaluate the relationship between residue and both PhAMPC and UES Duration, Spearman rank-order correlations were performed.

## Results

### Participants

The OPC cohort had an average age of 63.4 years (range: 49–78 years). Tumor locations included the base of tongue, tonsils, and palate, ranging in severity from T1 to T3 and N0 to N2c (Amin et al., 2018), with positive or negative Human papillomavirus status. All OPC participants had undergone conventional or accelerated radiation therapy (with or without chemotherapy) and were within the 3- to 6-month posttreatment time frame at the time of data collection. Additional details regarding the OPC cohort can be found in Table 2. The 12 male controls had an average age of 54.5 years (range: 39–70 years), with an average age difference between the OPC and control groups of 11 years.

**Table 2.** Oropharyngeal cancer (OPC) cohort demographics.

Participant	Site	Stage	Radiation dose (cGy)	Fractions	Chemotherapy?	Days post-RT
OPC 1	Base of tongue	T2, N2b	5,200	26	No	105
OPC 2	Base of tongue	T3, N2b	7,000	35	Yes	111
OPC 3	Base of tongue	T2, N2b	7,000	35	Yes	113
OPC 4	Base of tongue	T3, N2b	7,000	35	Yes^a^	113
OPC 5	Oropharynx	T0, N2b	7,000	35	Yes^a^	151
OPC 6	Soft palate	T3, N2b	7,000	35	Yes	140
OPC 7	Soft palate	T1, N0	6,000	35	No	134
OPC 8	Right tonsil	T2, N1	7,000	35	No	92
OPC 9	Base of tongue	T1, N2b	7,000	35	No	89
OPC 10	Right tonsil/base of tongue	T2, N2c	7,000	35	Yes	111
OPC 11	Base of tongue	T3, N2b	7,000	35	Yes	135
OPC 12	Left tonsil	T1, N2b	7,000	35	Yes	203

a Participants who had one to two chemotherapy sessions but discontinued due to adverse reactions. RT= Radiation Therapy.

### Sip Volume

Sip volumes in the OPC cohort averaged 16 ml, with a 95% confidence interval (CI) of 11.1–20.7 ml. Comparable sip volumes were found in the control group (*M* = 17 ml, 95% CI [12.7, 21.3]).

### Swallowing Safety

Penetration–aspiration events of concern (i.e., at least one PAS score ≥ 3) were found in nine of 12 participants in the oropharyngeal cancer cohort and in one healthy control (Wald χ^2^ = 7.99, *p* < .02, *OR* = 33, 95% CI [2.9, 371.31]). Incomplete LVC was found in six of 12 participants in the OPC cohort and in one healthy control (Wald χ^2^ = 5.27, *p* = .07, *OR* = 11, 95% CI [1.06, 114.09]). We found a statistically significant association between safe/impaired PAS scores and LVC integrity as assessed by Fisher's exact test (*p* < .001). Of the swallows displaying impaired safety (*n* = 10), seven (70%) occurred in the context of incomplete LVC; by contrast, LVC was documented as complete in 100% of safe swallows. Individuals with complete LVC had dramatically lower odds (*OR* = 0.18, 95% CI [0.06, 0.5]) of a PAS score of concern.

**Figure 1.**
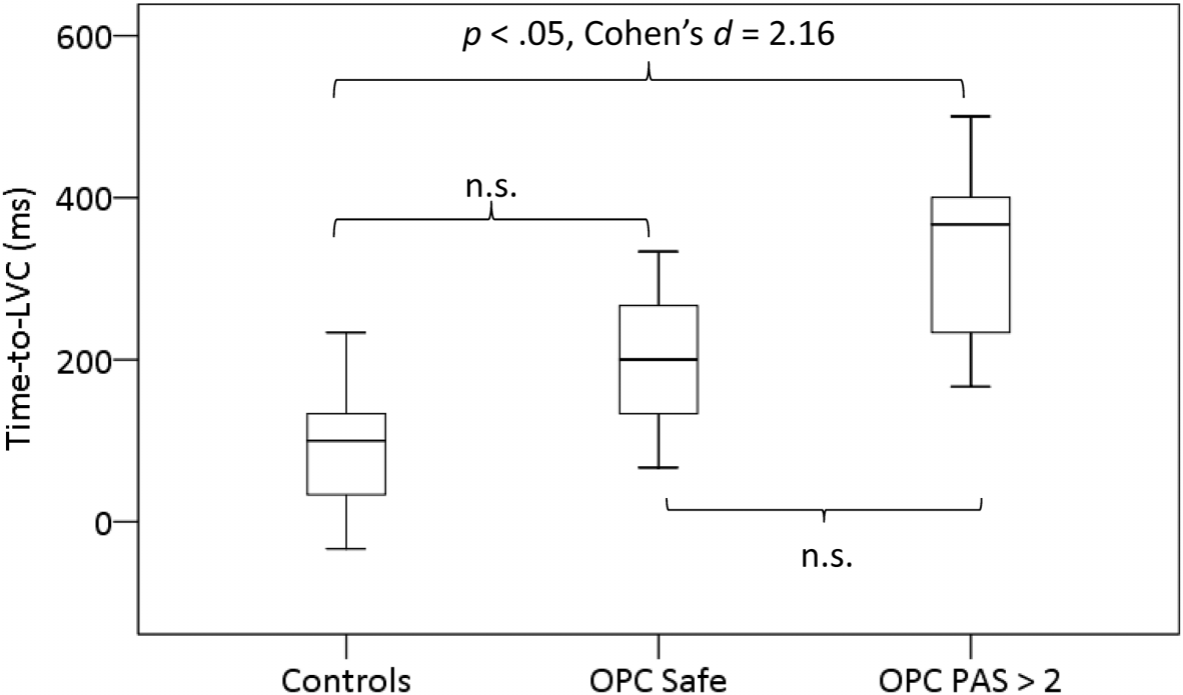
Group differences in time-to-LVC (in milliseconds). LVC = laryngeal vestibule closure; OPC = oropharyngeal carcinoma; PAS = Penetration–Aspiration Scale; n.s. = not significant.

Descriptive statistics for parameters related to swallowing safety are summarized by group in Table 3. Time-to-LVC was significantly longer in the OPC patients compared to the healthy controls, *F*(1, 21) = 20.074, *p* < .0001. Post hoc Sidak tests were conducted between three subgroups within the data: healthy participants, OPC participants with PAS scores of < 3 (*n* = 3), and OPC participants with PAS of ≥ 3 (*n* = 9). Group differences are illustrated in Figure 1. Time-to-LVC for the OPC safe swallows did not differ significantly from the healthy control swallows (*p* = .319) or from the OPC swallows with impaired safety (*p* = .227). However, the pairwise difference between healthy participant swallows and the impaired OPC swallows was statistically significant (*p* < .001) and had a large effect size (Cohen's *d* = 2.16). No significant differences in LVCDur were found between participant groups.

**Table 3.** Timing measures for oropharyngeal cancer (OPC) and healthy control participants.

Parameter	Group	Subgroup	*M*	*SD*	LCI	UCI
Time-to-LVC (ms)^a^	OPC	Safe (*n* = 3)	200	134	−131	532
		Unsafe (*n* = 9)	326	121	233	419
		Total	295	131	211	378
	Controls		91	78	39	143
LVCDur (ms)^a^	OPC		509	186	391	627
	Controls		552	116	474	630

*Note.* LCI = 95% confidence interval lower boundary; UCI = 95% confidence interval upper boundary; time-to-LVC = interval between the frame of hyoid burst onset and the frame of laryngeal vestibule closure; LVCDur = laryngeal vestibule closure duration.

a Timing measures were calculated in frames and converted to milliseconds using a formula of 29.975 frames per second.

### Swallowing Efficiency

The oropharyngeal cancer group had a mean total residue measurement of 2.9% (C2–4)^2^ (95% CI [0.3%, 5.6%]) compared to a mean of 2.3% (95% CI [0.6%, 4.1%]) in the control group, *H*(1) = 0.014, *p* = .907. Pharyngeal residue of concern (i.e., > 3% (C2–4)^2^) was found in four of 12 oropharyngeal cancer participants. However, three of 12 healthy control participants also displayed residue above the 3% (C2–4)^2^ threshold. The frequency of above-threshold residue did not differ significantly between groups (Wald χ^2^ = 3.997, *p* = .136); however, the odds were higher in the oropharyngeal cancer group (*OR* = 1.5, 95% CI [0.25, 8.84]).

Descriptive statistics for parameters related to swallowing efficiency are summarized by group in Table 4. The median for PhAR in the oropharyngeal cancer patients (53%) fell below the values seen in the healthy participants (*x̅* = 58%); however, this difference was not statistically significant, *H*(1) = 1.76, *p* = .184. A single participant in the entire data set, an oropharyngeal cancer patient, displayed unusually small PhAR below 36% of the (C2–4)^2^ reference scalar. Interestingly, however, five of 12 oropharyngeal cancer patients (i.e., 42%) had PhAR below the first quartile boundary seen in healthy young participants (i.e., 47% of the (C2–4)^2^ reference scalar) compared to zero of 12 participants in the healthy control group (Fisher's exact test, *p* = .04, *OR* = 1.714, 95% CI [1.06, 2.77]). The analyses did not reveal any apparent relationship between small PhAR measures (< 47% (C2–4)^2^) and above-normal total residue (Fisher's exact test, *p* = 1.0).

**Table 4.** Pixel-based measures for oropharyngeal cancer (OPC) and healthy control participants.

Parameter	Group	*M*/*Mdn* ^a^	*SD*/IQR^a^	LCI/Q1^a^	UCI/Q3^a^
PhAR %(C2–4)^2^	OPC	52.9^a^	27.1^a^	41.6^a^	68.7^a^
	Controls	58.4^a^	16.8^a^	54.8^a^	71.6^a^
PhAMPC %(C2–4)^2^	OPC	2.4^a^	7.6^a^	0.0^a^	7.6^a^
	Controls	1.5^a^	2.0^a^	0.0^a^	2.0^a^
UES diameter %(C2–4)^2^	OPC	21	7.5	15.6	26.4
	Controls	21.3	4.6	18.3	24.2
UESDur (ms)^a^	OPC	556	97	494	618
	Controls	503	59	465	541
Total residue %(C2–4)^2^	OPC	1.2^a^	3.4^a^	0.3^a^	3.8^a^
	Controls	1.77^a^	3.7^a^	0.0^a^	3.7^a^

*Note.* LCI = 95% confidence interval lower boundary; UCI = 95% confidence interval upper boundary; IQR = interquartile range; Q1 = first quartile; Q3 = third quartile; PhAR = pharyngeal area at rest; PhAMPC = pharyngeal area at maximum constriction; UES = upper esophageal sphincter; UESDur = duration of UES opening.

a Timing measures were calculated in frames and converted to milliseconds using a formula of 29.975 frames per second.

**Figure 2.**
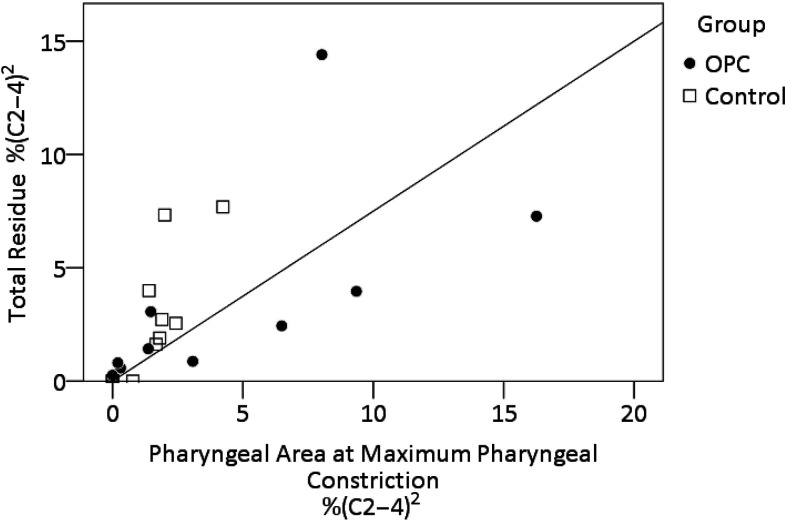
Relationship between pharyngeal area at maximum constriction and postswallow residue. OPC = oropharyngeal carcinoma.

The median PhAMPC for the oropharyngeal cancer participants measured 2.4% of the (C2–4)^2^ space (first quartile: 0%, third quartile: 7.6%), compared to median values in the healthy controls of 1.5% (first quartile: 0%, third quartile: 2%). The group difference in PhAMPC was not significant, *H*(1) = 0.492, *p* = .483. Of the 24 swallows in the data set, five (21%) displayed poor pharyngeal constriction (≥ 4% of (C2–4)^2^); four of these five cases came from the oropharyngeal cancer cohort (Wald χ^2^ = 7.08, *p* < .01, *OR* = 5.5, 95% CI [0.59, 59.01]). Overall, there was a strong, positive correlation between PhAMPC and the presence of residue, *r_s_*(24) = .859, *p* < .001, as illustrated in Figure 2. When binary classifications of above-normal PhAMPC (i.e., ≥ 4% (C2–4)^2^) were cross-tabulated with above-normal residue, a significantly greater proportion (80%) of the poor constriction cases displayed above-threshold residue (Fisher's exact test, *p* < .05, *OR* = 21.3, 95% CI [1.73, 263.67]). No significant differences were found between groups for measures of UES opening diameter, *F*(1, 20) = 0.010, *p* = .920, or duration, *F*(1, 22) = 2.592, *p* = .122, and there were no significant correlations between UES opening diameter and residue, *r_s_*(24) = .155, *p* = .492, or between UES opening duration and residue, *r_s_*(24) = .369, *p* = .076.

## Discussion

The purpose of this study was to characterize swallow physiology in a sample of oropharynx cancer patients who had undergone (chemo)radiotherapy for their cancer. We explored the relationships between swallowing physiology and functional measures of swallowing safety and efficiency on thin liquids. Additionally, we compared swallow timing and kinematics in these patients to a group of healthy controls.

Prolonged time-to-LVC was found to be a primary contributor to penetration–aspiration in this sample. Additionally, incomplete vestibule closure appears to be a key issue, given that all safe swallows in our data set were characterized by complete closure. These findings of incomplete LVC and time-to-LVC are consistent with those reported by Starmer et al. (2015), who found absent LVC or longer time-to-LVC to be associated with increased aspiration. These findings also point to the potential importance of compensatory techniques intended to facilitate complete and timely LVC, such as the chin-down maneuver (Young et al., 2015) or the use of texture modified liquids (Steele et al., 2019).

By contrast, our explorations did not reveal any group differences in LVCDur or any association between LVCDur and airway invasion. This finding differs from a previous report by Starmer et al. (2015), who observed shorter LVCDur in patients who presented with unsafe PAS scores (≥ 3). The mean duration of LVC in unsafe patients in the Starmer et al. study was reported to be 117 ms, while the participants in our study with PAS scores of ≥ 3 had much longer durations of closure, with a mean value of 467 ms. Differences in protocol warrant caution when comparing these two studies. Starmer's analyses included both 3 ml and cup-sips of thin, while our study analyzed cup-sips only. The inclusion of a smaller bolus size may have contributed to differences in LVCDur (Starmer et al., 2015). The LVCDurs in our study are similar to those from a study by Pauloski et al. (1998), in which participants presented a mean LVCDur of 460 ms across pooled thin and thick boluses.

Our results showed that participants in the oropharyngeal cancer group were significantly more likely to display poor pharyngeal constriction than those in the control group. Other studies have also reported impaired pharyngeal constriction in HNC patients (Kendall et al., 1998; Starmer et al., 2014). We postulate that radiation damage to pharyngeal tissue in the form of either edema or fibrosis, with associated changes in sensation and possible nerve damage, contributes to inefficient movement of musculature necessary for swallowing, especially soon after radiation (Pauloski et al., 1998; Popovtzer et al., 2009). Reduced pharyngeal constriction has been identified as a predictor of pharyngeal residue in patients with oculopharyngeal muscular dystrophy (Waito, Steele et al., 2018), amyotrophic lateral sclerosis (Waito, Tabor-Gray, et al., 2018), and neurogenic dysphagia (Stokely et al., 2015). However, despite worse pharyngeal constriction, our analyses did not find increased residue frequency or severity in the OPC group. The small sample size may have limited our ability to detect differences in residue severity across groups. Furthermore, given that our analysis was limited to thin liquid tasks, the possibility that worse constriction might be seen in the context of thicker bolus consistencies, thereby contributing to greater residue, and worse swallowing efficiency is not fully explored.

It has been suggested that post-RT edema may also impact other measures; however, the participants with oropharyngeal cancer did not display marked restrictions in PhAR compared to age-matched controls, and we did not find any relationship between PhAR and measures of pharyngeal residue. It is important to consider age-related differences between healthy young and healthy older participants. Our healthy comparison cohort was heavily populated by older males, in whom dilated PhAR has been previously observed (Leonard et al., 2004b; Molfenter et al., 2018).

Similarly, our data did not demonstrate any relationships between measures of UES diameter or opening duration and pharyngeal residue. Eisbruch et al. (2002) reported reduced UES opening in their patients and associated these findings with an increased risk of aspiration during the swallow. However, objective measures of UES diameter were not reported, and the timing of penetration–aspiration events relative to LVC or UES opening was not described. Pauloski et al. (1998) found increased pharyngeal residue and shorter UESDur in patients who had undergone RT; however, our study failed to find similar results. Further research is required to confirm whether reductions in UES diameter or opening duration relate either to swallowing safety or to pharyngeal residue, especially in HNC participants who are prone to cricopharyngeal bar and dysfunction (Queija et al., 2009).

### Limitations

It is important to acknowledge the limitations of this study. Our study was conducted with a small sample of oropharyngeal cancer patients, and the sample was comprised of male participants exclusively. Due to the goal of achieving homogeneity of tumor location within our sample, participant accrual was a laborious process. Stopping rules resulted in fewer than three thin liquid boluses for three of the OPC participants; consequently, our analysis was based on the worst swallowing safety example observed per participant and did not allow consideration of the frequency of penetration–aspiration across repeated boluses. The descriptions of swallowing mechanics were made on thin liquid swallows only and did not incorporate variations in bolus size as a covariate. Raters were blinded to participant but not by group (healthy vs. OPC). Additionally, all data were analyzed at the participant level, rendering it difficult to compare to previous studies with data reported at the bolus level.

## Conclusions

In this study, we identified various mechanisms that contribute to impaired swallowing safety and efficiency on thin liquid swallows in a group of 12 oropharyngeal cancer patients, compared to 12 healthy controls. Our data demonstrate the importance of the integrity and timing of LVC for airway protection. These mechanisms were significantly impaired in our patient cohort within the 3- to 6-month time frame post-RT for OPC. Dysphagia in our oropharyngeal cancer cohort was also characterized by reduced pharyngeal constriction and associated pharyngeal residue. While our analyses point to key pathophysiological components of swallowing following (chemo)radiotherapy, larger longitudinal studies are warranted to investigate the progression of these mechanisms over time. Additional research exploring swallowing in larger groups of HNC participants across various bolus consistencies is recommended in order to expand the available knowledge regarding swallowing in this patient population.

## Author Contributions

**Douglas B. Chepeha:** Methodology (Supporting), Supervision (Supporting), Writing–Review & Editing (Supporting). **Douglas B. Chepeha:** Methodology (Supporting), Supervision (Supporting), Writing–Review & Editing (Supporting). **Andrew J. Hope:** Methodology (Supporting), Supervision (Supporting), Writing–Review & Editing (Supporting). **Melanie Peladeau-Pigeon:** Data curation (Supporting), Writing–Review & Editing (Supporting). **Ashley A. Waito:** Conceptualization (Supporting), Formal analysis (Supporting), Writing–Review & Editing (Supporting). **Catriona M. Steele:** Conceptualization (Equal), Data curation (Equal), Formal analysis (Equal), Funding acquisition (Lead), Methodology (Equal), Resources (Lead), Software (Lead), Supervision (Supporting), Writing–Original Draft (Supporting), Writing–Review & Editing (Equal).

## Author Contributions

- Carly E. A. Barbon was the principal investigator for the project and was responsible for project design, participant recruitment, data collection, statistical analysis, and manuscript writing.
- Douglas B. Chepeha and Andrew J. Hope contributed to study design, participant recruitment, and manuscript editing.
- Melanie Peladeau-Pigeon assisted with data processing, data management, and manuscript editing.
- Ashley A. Waito assisted with data analysis and manuscript editing.
- Catriona M. Steele supervised the project and contributed to study design, statistical analysis, and manuscript writing and editing.

## References

[bib1] Amin, M. B. , Greene, F. L. , Edge, S. , Schilsky, R. L. , Gaspar, L. E. , Washington, M. K. , Sullivan, D. C. , & Brookland, R. K. (2018). American Joint Committee on Cancer (AJCC) cancer staging manual (8th ed.). Springer.

[bib2] Ardran, G. M. , & Kemp, F. H. (1952). Some aspects of the mechanism of swallowing. Gastroenterologia, 78(6), 347–349. https://doi.org/10.1159/000199701 13021322 10.1159/000199701

[bib3] Ardran, G. M. , & Kemp, F. H. (1967). The mechanism of the larynx. II. The epiglottis and closure of the larynx. The British Journal of Radiology, 40(473), 372–389. https://doi.org/10.1259/0007-1285-40-473-372 6022785 10.1259/0007-1285-40-473-372

[bib4] Dejaeger, E. , Pelemans, W. , Ponette, E. , & Joosten, E. (1997). Mechanisms involved in postdeglutition retention in the elderly. Dysphagia, 12(2), 63–67. https://doi.org/10.1007/PL00009520 9071804 10.1007/PL00009520

[bib5] Eisbruch, A. , Lyden, T. , Bradford, C. R. , Dawson, L. A. , Haxer, M. J. , Miller, A. E. , Teknos, T. N. , Chepeha, D. B. , Hogikyan, N. D. , Terrell, J. E. , & Wolf, G. T. (2002). Objective assessment of swallowing dysfunction and aspiration after radiation concurrent with chemotherapy for head-and-neck cancer. International Journal of Radiation Oncology, Biology, Physics, 53(1), 23–28. https://doi.org/10.1016/S0360-3016(02)02712-8 10.1016/s0360-3016(02)02712-8 12007937

[bib6] Eisbruch, A. , Schwartz, M. , Rasch, C. , Vineberg, K. , Damen, E. , Van As, C. J. , Marsh, B. , Pameijer, F. A. , & Balm, A. J. (2004). Dysphagia and aspiration after chemoradiotherapy for head-and-neck cancer: Which anatomic structures are affected and can they be spared by IMRT. International Journal of Radiation Oncology, Biology, Physics, 60(5), 1425–1439. https://doi.org/10.1016/j.ijrobp.2004.05.050 10.1016/j.ijrobp.2004.05.050 15590174

[bib7] Farwell, D. G. , Rees, C. J. , Mouadeb, D. A. , Allen, J. , Chen, A. M. , Enepekides, D. J. , & Belafsky, P. C. (2010). Esophageal pathology in patients after treatment for head and neck cancer. Otolaryngology—Head & Neck Surgery, 143(3), 375–378. https://doi.org/10.1016/j.otohns.2010.05.006 20723774 10.1016/j.otohns.2010.05.006

[bib8] Inamoto, Y. , Fujii, N. , Saitoh, E. , Baba, M. , Okada, S. , Katada, K. , Yasunori, O. , & Palmer, J. B. (2011). Evaluation of swallowing using 320-detector-row multislice CT. Part II: Kinematic analysis of laryngeal closure during normal swallowing. Dysphagia, 26(3), 209–217. https://doi.org/10.1007/s00455-010-9276-2 20204412 10.1007/s00455-010-9276-2

[bib9] Isitt, J. (2006). Oral mucositis (OM) related morbidity and resource utilization in a prospective study of head and neck cancer (HNC) patients. Journal of Clinical Oncology, 24(18_suppl), 5539–5539. https://doi.org/10.1200/jco.2006.24.18_suppl.5539

[bib10] Kahrilas, P. J. , Logemann, J. A. , Lin, S. , & Ergun, G. A. (1992). Pharyngeal clearance during swallowing: A combined manometric and videofluoroscopic study. Gastroenterology, 103(1), 128–136. https://doi.org/10.1016/0016-5085(92)91105-D 1612322 10.1016/0016-5085(92)91105-d

[bib11] Kendall, K. A. , McKenzie, S. W. , Leonard, R. J. , & Jones, C. (1998). Structural mobility in deglutition after single modality treatment of head and neck carcinomas with radiotherapy. Head & Neck, 20(8), 720–725. https://doi.org/10.1002/(SICI)1097-0347(199812)20:8<720::AID-HED10>3.0.CO;2-L 9790294 10.1002/(sici)1097-0347(199812)20:8<720::aid-hed10>3.0.co;2-l

[bib12] Koo, T. K. , & Li, M. Y. (2016). A guideline of selecting and reporting intraclass correlation coefficients for reliability research. Journal of Chiropractic Medicine, 15(2), 155–163. https://doi.org/10.1016/j.jcm.2016.02.012 27330520 10.1016/j.jcm.2016.02.012 PMC4913118

[bib13] Kotz, T. , Abraham, S. , Beitler, J. J. , Wadler, S. , & Smith, R. V. (1999). Pharyngeal transport dysfunction consequent to an organ-Sparing protocol. Archives of Otolaryngology—Head & Neck Surgery, 125(4), 410–413. https://doi.org/10.1001/archotol.125.4.410 10208678 10.1001/archotol.125.4.410

[bib14] Kotz, T. , Costello, R. , Li, Y. , & Posner, M. R. (2004). Swallowing dysfunction after chemoradiation for advanced squamous cell carcinoma of the head and neck. Head & Neck, 26(4), 365–372. https://doi.org/10.1002/hed.10385 15054740 10.1002/hed.10385

[bib15] Langmore, S. E. , & Krisciunas, G. P. (2010). Dysphagia after radiotherapy for head and neck cancer: Etiology, clinical presentation, and efficacy of current treatments. SIG 13 Perspectives on Swallowing and Swallowing Disorders (Dysphagia), 19(2), 32–38. https://doi.org/10.1044/sasd19.2.32

[bib16] Leonard, R. , Kendall, K. A. , & McKenzie, S. (2004a). UES opening and cricopharyngeal bar in nondysphagic elderly and nonelderly adults. Dysphagia, 19(3), 182–191. https://doi.org/10.1007/s00455-004-0005-6 15383948 10.1007/s00455-004-0005-6

[bib17] Leonard, R. , Kendall, K. A. , & McKenzie, S. (2004b). Structural displacements affecting pharyngeal constriction in nondysphagic elderly and nonelderly adults. Dysphagia, 19(2), 133–141. https://doi.org/10.1007/s00455-003-0508-6 15382802 10.1007/s00455-003-0508-6

[bib18] Logemann, J. A. , Kahrilas, P. J. , Cheng, J. , Pauloski, B. R. , Gibbons, P. J. , Rademaker, A. W. , & Lin, S. (1992). Closure mechanisms of laryngeal vestibule during swallow. American Journal of Physiology, 262(2 Pt. 1), G338–G344. https://doi.org/10.1152/ajpgi.1992.262.2.G338 10.1152/ajpgi.1992.262.2.G338 1539666

[bib19] Molfenter, S. M. , Lenell, C. , & Lazarus, C. L. (2018). Volumetric changes to the pharynx in healthy aging: Consequence for pharyngeal swallow mechanics and function. Dysphagia, 34(1), 129–137. https://doi.org/10.1007/s00455-018-9924-5 30039259 10.1007/s00455-018-9924-5 PMC6344328

[bib20] Murphy, B. A. , & Gilbert, J. (2009). Dysphagia in head and neck cancer patients treated with radiation: Assessment, sequelae, and rehabilitation. Seminars in Radiation Oncology, 19(1), 35–42. http://doi.org/10.1016/j.semradonc.2008.09.007 19028344 10.1016/j.semradonc.2008.09.007

[bib21] Nativ-Zeltzer, N. , Logemann, J. A. , & Kahrilas, P. J. (2014). Comparison of timing abnormalities leading to penetration versus aspiration during the oropharyngeal swallow. The Laryngoscope, 124(4), 935–941. https://doi.org/10.1002/lary.24408

[bib22] Pauloski, B. R. , Rademaker, A. W. , Logemann, J. A. , & Colangelo, L. A. (1998). Speech and swallowing in irradiated and nonirradiated postsurgical oral cancer patients. Otolaryngology—Head & Neck Surgery, 118(5), 616–624. https://doi.org/10.1177/019459989811800509 9591859 10.1177/019459989811800509

[bib24] Popovtzer, A. , Cao, Y. , Feng, F. , & Eisbruch, A. (2009). Anatomical changes in the pharyngeal constrictors after chemo-irradiation of head and neck cancer and their dose–effect relationships: MRI-based study. Radiotherapy & Oncology, 93(3), 510–515. https://doi.org/10.1016/j.radonc.2009.05.013 19520446 10.1016/j.radonc.2009.05.013 PMC2787803

[bib25] Queija, D. S. , Portas, J. G. , Dedivitis, R. A. , Lehn, C. N. , & Barros, A. P. (2009). Swallowing and quality of life after total laryngectomy and pharyngolaryngectomy. Brazilian Journal of Otorhinolaryngology, 75(4), 556–564. https://doi.org/10.1016/S1808-8694(15)30496-1 19784426 10.1016/S1808-8694(15)30496-1 PMC9446096

[bib26] Rofes, L. , Arreola, V. , Romea, M. , Palomera, E. , Almirall, J. , Cabré, M. , Serra-prat, M. , & Clavé, P. (2010). Pathophysiology of oropharyngeal dysphagia in the frail elderly. Journal of Neurogastroenterology and Motility, 22(8), 851–858. https://doi.org/10.1111/j.1365-2982.2010.01521.x 10.1111/j.1365-2982.2010.01521.x 20529208

[bib27] Rosenbek, J. C. , Robbins, J. A. , Roecker, E. B. , Coyle, J. L. , & Wood, J. L. (1996). A Penetration–Aspiration Scale. Dysphagia, 11(2), 93–98. https://doi.org/10.1007/BF00417897 8721066 10.1007/BF00417897

[bib28] Rosner, B. (2016). Fundamentals of biostatistics (8th ed.). Brooks/Cole Cengage Learning.

[bib29] Starmer, H. M. , Quon, H. , Kumar, R. , Alcorn, S. , Murano, E. , Jones, B. , & Humbert, I. (2015). The effect of radiation dose on swallowing: Evaluation of aspiration and kinematics. Dysphagia, 30(4), 430–437. https://doi.org/10.1007/s00455-015-9618-1 26025757 10.1007/s00455-015-9618-1

[bib30] Starmer, H. M. , Tippett, D. , Webster, K. , Quon, H. , Jones, B. , Hardy, S. , & Gourin, C. G. (2014). Swallowing outcomes in patients with oropharyngeal cancer undergoing organ-preservation treatment. Head & Neck, 36(10), 1392–1397. https://doi.org/10.1002/hed.23465 24038454 10.1002/hed.23465

[bib31] Steele, C. M. , Chak, V. , Dhindsa, A. , Draimin, R. , Nagy, A. , Peladeau-Pigeon, M. , Tapson, S. , Torreiter, S. , Wolkin, T. , & Waito, A. A. (2015). Timing plays a major role in the pathophysiology of aspiration. Dysphagia, 31, 258.

[bib32] Steele, C. M. , Peladeau-Pigeon, M. , Barbon, C. A. E. , Guida, B. T. , Namasivayam-MacDonald, A. M. , Nascimento, W. V. , Smaoui, S. , Tapson, M. S. , Valenzon, T. J. , Waito, A. , & Wolkin, T. S. (2019). Reference values for healthy swallowing across the range from thin to extremely thick liquids. Journal of Speech, Language, and Hearing Research, 62(5), 1338–1363. https://doi.org/10.1044/2019_JSLHR-S-18-0448 10.1044/2019_JSLHR-S-18-0448 PMC6808317 31021676

[bib33] Stokely, S. L. , Peladeau-Pigeon, M. , Leigh, C. , Molfenter, S. M. , & Steele, C. M. (2015). The relationship between pharyngeal constriction and post-swallow residue. Dysphagia, 30(3), 349–356. https://doi.org/10.1007/s00455-015-9606-5 25920993 10.1007/s00455-015-9606-5 PMC4469308

[bib34] Turcotte, M. C. , Herzberg, E. G. , Balou, M. , & Molfenter, S. M. (2018). Analysis of pharyngeal edema post-chemoradiation for head and neck cancer: Impact on swallow function. Laryngoscope Investigative Otolaryngology, 3(5), 377–383. https://doi.org/10.1002/lio2.203 30410991 10.1002/lio2.203 PMC6209611

[bib35] Ursino, S. , Seccia, V. , Cocuzza, P. , Ferrazza, P. , Briganti, T. , Matteucci, F. , Fatigante, L. , Giusti, P. , Grosso, M. , Locantore, L. , Morganti, R. , Nacci, A. , Fraceschini, S. , Pair, F. , Caramella, D. , & Fattori, B. (2016). How does radiotherapy impact swallowing function in nasopharynx and oropharynx cancer? Short-term results of a prospective study. Acta Otorhinolaryngologica Italica, 36(3), 174–184. https://doi.org/10.14639/0392-100X-640 27070541 10.14639/0392-100X-640 PMC4967765

[bib37] Viera, A. J. , & Garrett, J. M. (2005). Understanding interobserver agreement: The kappa statistic. Family Medicine, 37(5), 360–363. 15883903

[bib38] Vose, A. , & Humbert, I. (2018). “Hidden in plain sight”: A descriptive review of laryngeal vestibule closure. Dysphagia, 34(3), 281–289. https://doi.org/10.1007/s00455-018-9928-1 30062547 10.1007/s00455-018-9928-1 PMC6408979

[bib39] Waito, A. A. , Steele, C. M. , Peladeau-Pigeon, M. , Genge, A. , & Argov, Z. (2018). A preliminary videofluoroscopic investigation of swallowing physiology and function in individuals with oculopharyngeal muscular dystrophy (OPMD). Dysphagia, 33(6), 789–802. https://doi.org/10.1007/s00455-018-9904-9 29725764 10.1007/s00455-018-9904-9

[bib40] Waito, A. A. , Tabor-Gray, L. C. , Steele, C. M. , & Plowman, E. K. (2018). Reduced pharyngeal constriction is associated with impaired swallowing efficiency in amyotrophic lateral sclerosis (ALS). Journal of Neurogastroenterology and Motility, 30(12), e13450 https://doi.org/10.1111/nmo.13450 10.1111/nmo.13450 PMC6249041 30129164

[bib41] Young, J. L. , Macrae, P. , Anderson, C. , Taylor-Kamara, I. , & Humbert, I. A. (2015). The sequence of swallowing events during the chin-down posture. The American Journal of Speech-Language Pathology, 24(4), 659–670. https://doi.org/10.1044/2015_AJSLP-15-0004 26225454 10.1044/2015_AJSLP-15-0004 PMC4698467

